# Impact of UGT2B17 gene deletion on the steroid profile of an athlete

**DOI:** 10.14814/phy2.12645

**Published:** 2015-12-15

**Authors:** Pilar Martín‐Escudero, Jesús Muñoz‐Guerra, Nayade Del Prado, Mercedes Galindo Canales, Manuel Fuentes Ferrer, Soledad Vargas, Ana B. Soldevilla, Ester Serrano‐Garde, Francisco Miguel‐Tobal, Marisa Maestro de las Casas, Cristina Fernandez‐Pérez

**Affiliations:** ^1^ Madrid School of Sports MedicineFaculty of MedicineUniversidad Complutense de MadridMadridSpain; ^2^ Doping Control Laboratory of Madrid and Anti‐doping State AgencyMadridSpain; ^3^ Unit of Clinical Management (UGC)Department of Preventive MedicineHospital Clínico San CarlosMadridSpain; ^4^ Unit of Genomics of the Hospital Clínico San CarlosMadridSpain; ^5^ Institute of Healthcare Research of the Hospital Clínico San Carlos (IdISSC)MadridSpain

**Keywords:** Exercise, doping, UGT2B17 genotype, steroid profile

## Abstract

The measurement of the testosterone to epitestosterone ratio (T/E ratio) in urine is often used as a marker for testosterone administration in the doping control field. This study examines the frequencies of the different expression forms of the UGT2B17 gene, and assesses their effects on this marker in volunteer subjects. The sample for this descriptive study was composed of male and female athletes aged between 16 and 55 years old who practiced different sports disciplines. All participants underwent a sports‐medical physical examination, and subsequently provided 10 urine samples consecutively over a period of 48 h. The dependent variable examined was T/E and the main independent variable was the UGT2B17 gene polymorphism. During 1 year, 1410 urine samples were obtained from 141 athletes. The frequencies of the three genotypes were as follows: wt homozygotes (*ins/ins*) 48.2% (*n* = 68), mutant homozygotes (*del/del*) 12.1% (*n* = 17), and heterozygotes (*ins/del*) 39.7% (*n* = 56). Genotype distributions varied significantly (*P* < 0.001) according to ethnicity, 80% of Asian subjects being homozygous for the gene deletion (*del/del*) compared to 6.9% of Caucasian subjects. A multivariate analysis adjusted for genotype, age, sex, and sports discipline revealed that athletes with the *del/del* polymorphism showed a significantly lower mean T/E than heterozygotes (*ins/del*). In contrast, homozygous athletes for the gene insertion (*ins/ins)* showed higher mean T/E ratios than heterozygotes (*ins/del*). UGT2B17 gene deletion has a strong influence on the T/E ratio in urine, which is the most efficient indicator of testosterone prohormone misuse. Others factors studied seem not to have such an impact. The genotyping of UGT2B17 is an important source of information for understanding steroid profiling in the doping control field; therefore it is suggested that it be included in the Athletes Biological Passport.

## Introduction

Practicing a sport has several health benefits which are put at risk when athletes use performance‐enhancing drugs included in the WADA list of forbidden substances. Doping puts the athlete's health at risk, violates the rights of their competitors and goes against the concept of fair play (Baron et al. 2007).

In the beginning antidoping controls aimed at detecting the intake of steroids, and among them the consumption of testosterone and/or its precursors. Some decades ago the main indicator of testosterone misuse was a testosterone to epitestosterone ratio (T/E) in urine greater than 6 (Donike et al. 1993; Juul et al. 2009). In 2004, the World Anti‐Doping Agency decided to decrease this threshold to 4 (Juul et al. 2009; Sottas et al. 2011; National Institute on Drug Abuse).

Testosterone is mainly excreted as a glucuronide conjugate after its metabolism by uridine diphosphate (UDP) and glucuronosyl transferase (UGT). UGT2B7, UGT2B15, and UGT2B17 are known to be the main glucuronidation catalysts of androgens and their metabolites in humans (Schulze et al. 2008). Testosterone is mainly conjugated by UGT2B17 and to a lesser extent by UGT2B15. The main androgen substrate of UGT2B15 is androstane‐3*α* 17*β*‐diol. The actions of UGT2B17 are 96% in common with those of UGT2B15. The enzyme UGT2B7 also has the capacity to conjugate epitestosterone, while testosterone is a poor substrate for this enzyme. It has been established that a deletion polymorphism in the gene that codes for UGT2B17 (Gallagher et al. 2007b) correlates highly with testosterone levels in urine (Jakobsson et al. 2006). Thus, subjects lacking this gene have been found to show a T/E ratio lower than 0.4 (Jakobsson et al. 2006; Gallagher et al. 2007a, 2007b; Strahm et al. 2007). This polymorphism is much more common in Asian subjects than Caucasian subjects and its prevalence has been estimated at 66.7% in Asians versus 9.3% in Caucasians (Schulze et al. 2008; Strahm et al. 2009).

This study sought to determine the distributions of expression forms of the UGT2B17 gene in athletes and how the T/E ratio is affected by this factor.

## Methods

### Study design and population

The study design was descriptive with the prospective collection of data. The subjects included were male and female athletes aged 16–55 years from different sports disciplines. Participants had maintained a high competition level over at least 3–5 years and trained 2–3 hours per day on 3–5 days per week. The study protocol received the approval of the Ethics and Clinical Research Committee of the Hospital Clínico San Carlos (HCSC), Madrid, Spain. All participants or their legal guardians if the athlete was under 18 signed an informed consent form. Athletes were invited to participate via the medical services of their national sports federations.

All the athletes underwent a sports‐medical study. This study included a questionnaire designed to determine the lifestyle, sociodemographics, and sports characteristics of the subjects, a blood test, anthropometric measurements, and a nutrition questionnaire. Ten consecutive urine samples were collected from each participant, over 48 h, interrupted at night, at the following time points: 9 pm–10 pm (Day 1), first urine upon waking (Day 1 and Day 2), 10 am–11 am (Day 1 and Day 2), 1 pm–2 pm (Day 1 and Day 2), 5 pm–6 pm (Day 1 and Day 2).

**Figure 1:**
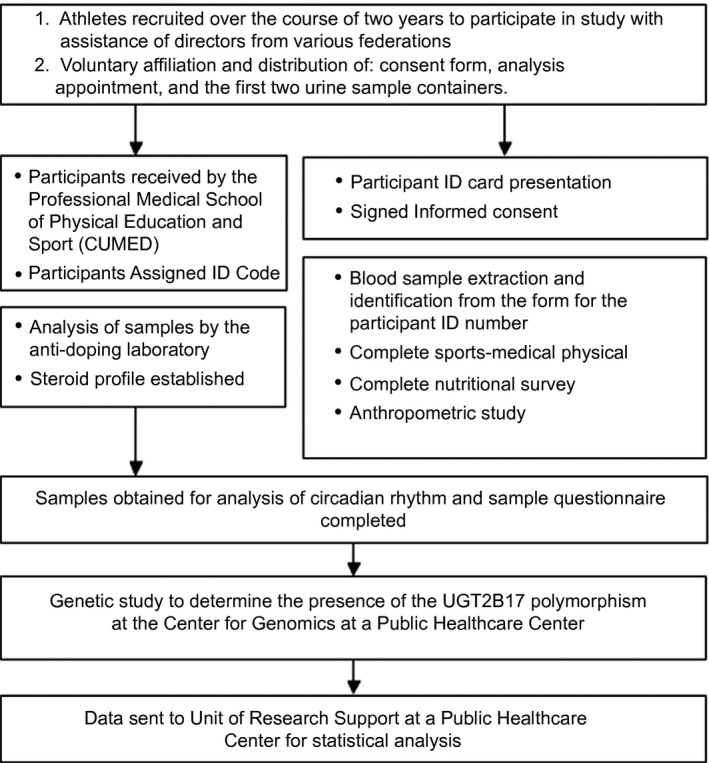
Data collection process.

Details of the data collection process are provided in Figure 1.

The dependent variable in this study was the T/E ratio. The main independent variable was the UGT2B17 gene polymorphism, but some other factors were assessed in order to determine if they could affect the T/E ratio, among them; type of sport, gender, age, etc. The presence in an individual of both alleles (insertion, deletion) indicates a heterozygous genotype. The presence of the wild‐type (wt) allele and lack of the mutant allele indicates a wt homozygous genotype. A mutant allele in the absence of the wt allele indicates a mutant homozygous genotype. The remaining independent variables were as follows: age, sex, sports discipline, type (anaerobic, mixed, and aerobic), ethnicity (Caucasian, Asian or other), civil state (single, married/partner, or separated/divorced), level of education, and sports discipline.

#### Urine steroid profiles

A validated screening procedure implemented at the Madrid Anti‐doping Laboratory belonging to AEPSAD was used to determine urine levels of testosterone, epitestosterone, androsterone (A), etiocholanolone (Etio), 5*α*‐androstan‐3*α*,17*β*‐diol (5*α*‐diol), and 5*β*‐androstan‐*α*,17*β*diol (5*β*‐diol). This method is based on several consecutive steps that include; liquid phase extraction at alkaline pH using diethyl ether (after previous urine sample purification with C‐18 cartridges) followed by enzyme hydrolysis by *E. coli* enzyme. The final extract was dried and TMS derivatized and a 2 *μ*L‐aliquot injected into a gas chromatograph–mass spectrometer system (GCMS 5973 Agilent). All urinary values are expressed as the unconjugated plus the glucuronide conjugated fraction. To calculate steroid concentrations, the system was previously calibrated using an external calibration mixture. Deuterated internal standards of each of the six steroids were added to each sample. The effect of urine dilution was corrected by normalizing the samples to a specific gravity of 1.020.

### Genotyping

Genomic DNA was extracted from each subject using the QIAamp^®^ DNA Blood Mini kit (Qiagen), quantified by spectrophotometry and diluted to the concentration necessary for genotyping for the UGT2B17 deletion polymorphism. Genotyping was performed by polymerase chain reaction analysis as described by Schulze et al. (2009).

### Statistical analysis

Qualitative variables are provided with their frequency distributions. Quantitative variables are expressed as their mean and standard deviation (SD) and variables not showing a normal distribution as medians and interquartile ranges (IQR = P25–P75). Associations between qualitative variables were assessed using the chi‐squared or Fisher's exact test when more than 25% of expected values were less than 5. Comparisons of continuous variables for the T/E ratio and the independent variables examined were performed via a nonparametric median test. An analysis was performed on all the urine samples collected (*n* = 1410). A linear regression model was fitted and adjusted using generalized estimating equations (GEE) to control for intrasubject variability. The observations made on the athletes were grouped into clusters of data showing intragroup correlations, but which were independent among the different groups. Given that the dependent variable in our model (T/E) showed a marked positively skewed distribution, the data were log transformed. The model's *β* coefficients were transformed using the antilogarithmic function and were interpreted as ratios of means relative to the reference category of each independent variable.

All statistical tests were performed using STATA 11.0. software. Significance was set at *P* < 0.05.

## Results

The recruitment period lasted 12 months starting in September 2010 and ending in August 2011. During this period, a total of 140 athletes were involved in the study. The sociodemographic description of the sample is shown in Table 1. In summary, the mean age was 29.3 years old (SD: 11.5), 62.4% were men and 7.1% were Asian. The most common sports practiced among the volunteers were basketball, mountain races, and rugby, in total these disciplines accounted for 58.1% of the participants. From the physiological point of view, the type of sports discipline was distributed in 46.1% mixed, 20.6% anaerobic, and the rest aerobic.

**Table 1:** Sociodemographic description of the population submitted to the study

	*n* (%)
Mean age	29.3 (11.5)
Gender	
Male	88 (62.4)
Female	53 (37.6)
Ethnicity	
Caucasian	130 (92.2)
Asian	10 (7.1)
Black	1 (0.7)
Classification of sports according to oxygen debt	
Anaerobic	29 (20.6)
Mixed	65 (46.1)
Aerobic	47 (33.3)
Sports disciplines	
Basketball	41 (29.1)
Mountain races	25 (17.7)
Rugby	16 (11.3)
Cycling	11 (7.8)
Weightlifting	9 (6.4)
Volleyball	8 (5.7)
Martial arts	8 (5.7)
Triathlon	5 (3.5)
Athletics	5 (3.5)
Olympic wrestling	4 (2.8)
Others	9 (6.4)

### Genetic study

Distributions of the athletes according to the genotypes examined were as follows: 48.2% (68) wt homozygous (*ins/ins*), 12.1% (Sottas et al. 2007) mutant genotype homozygous (*del/del*), and 39.7% (56) heterozygous (*ins/del*). When genotype distributions were examined by ethnicity it was observed that 9 (6.9%) of the Caucasian athletes showed the gene mutation (del/del) compared to 8 (80.0%) of the Asian athletes, the difference being significant (*P* < 0.001). None of the Asian athletes showed the *ins/del* genotype, whereas 68 (52.3%) of the Caucasians did feature this mutation. Table 2 shows demographic and sports variable distributions by genotype. Athletes homozygous for the mutant allele (*del/del*) were significantly older, were mainly Asian and more often, though not significantly, practiced an anaerobic sport.

**Table 2:** Individual characteristics by UGT2B17 genotype

	Heterozygote (*ins/del*) *n* %	Mutant homozygote (*del/del*) *n* %	Wt homozygote (*ins/ins*) *n* %	*P*
Gender				
Male	35 (62.5)	11 (64.7)	42 (61.8)	0.975
Female	21 (37.5)	6 (32.3)	26 (38.2)	
Age (years) quartiles				
<21	15 (26.8)	4 (23.5)	22 (32.3)	0.033
21–27	15 (26.8)	2 (11.8)	13 (19.1)	
28–35	15 (26.8)	1 (5.9)	20 (29.4)	
>35	11 (19.6)	10 (58.8)	13 (19.1)	
Ethnicity				
Caucasian	5 (96.6)	9 (52.9)	68 (100.0)	<0.001
Asian	2 (3.6)	8 (47.1)	0 (0.0)	
Black	1 (1.8)	0 (0.0)	0 (0.0)	
Oxygen debt				
Anaerobic	10 (17.9)	8 (47.1)	11 (16.2)	0.072
Mixed	25 (44.6)	4 (23.5)	36 (52.9)	
Aerobic	21 (37.5)	5 (29.4)	21 (30.9)	

### Univariate analysis of the relationship between sociodemographic, sports, or genetic variables and the T/E ratio

For the 1410 urine samples collected from the 140 athletes, the median T/E ratio was 0.99 (IQR: 0.57–1.52). Median testosterone and epitestosterone concentrations were 8.5 ng/mL (IQR: 3.3–23.8) and 14.6 ng/mL (IQR: 6.5–29.4), respectively.

**Figure 2:**
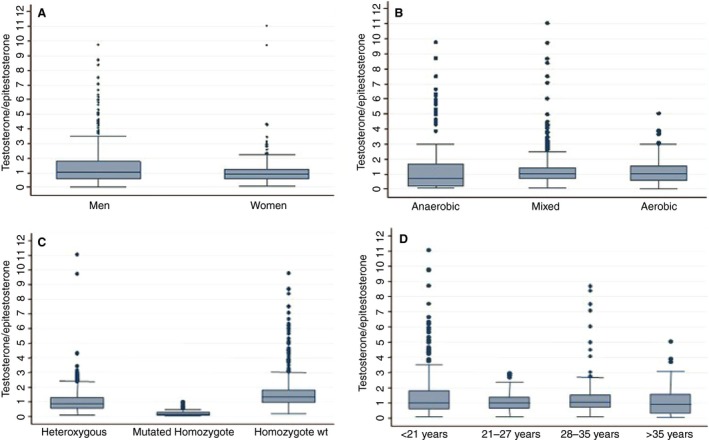
Testosterone/epitestosterone ratio according to demographic, sports, and genetic variables.

Figure 2 shows box diagrams of the T/E index variable according to gender, sports discipline type, age (by quartiles), and genotype for the UGT2B17 gene. In men, the median T/E ratio (1.08; IQR: 0.56–1.79) was significantly greater (*P* < 0.001) than in women (0.92; IQR: 0.58–1.25). Median T/E was 0.71 (IQR: 0.24–1.69) in athletes undertaking anaerobic sports, 1.01 (IQR: 0.71–1.45) for sports eliciting a mixed anaerobic/aerobic metabolism and 1.06 for aerobic sports (IQR: 0.59–1.57), significant differences being observed between the three categories (*P* < 0.001). Median T/E ratios for the four age categories did not significantly vary (*P* = 0.261). Individuals with the *del/del* genotype showed significantly lower T/E ratios (0.14; IQR: 0.1–0.26) (*P* < 0.001) than *ins/del* (0.86; IQR: 0.55–1.29) or *ins/ins* (1.30; IQR: 0.94–1.78) individuals.

### Multivariate analysis of the relationship between sociodemographic, sport, or genetic variables and the T/E ratio

Table 3 shows the data obtained in the multiple linear regression model. The only variable independently related to T/E was genotype. Athletes homozygous for the mutant allele (*del/del*) showed an 83% lower mean T/E than heterozygotes (*ins/del*), whereas in homozygous (*ins/ins)* individuals, the mean T/E was 53% higher than in the *ins/del* subjects.

**Table 3:** Multiple linear regression model (GEE). Relationship between gender, age, oxygen debt of sport and UGT2B17 genotype with urine testosterone/epitestosterone ratio

	Ratio of means	Lower limit 95% CI	Upper limit 95% CI	*P*
Gender				
Male	1			
Female	0.89	0.71	1.12	0.325
Age (years) quartiles				
<21	1			
21–27	0.89	0.66	1.19	0.429
28–35	0.89	0.65	1.20	0.439
>35	1.08	0.74	1.58	0.685
UGT2B17 polymorphism				
Heterozygote	1			
Mutant homozygote	0.17	0.12	0.25	<0.0001
Wt homozygote	1.53	1.22	1.91	<0.001
Sport according oxygen debt				
Anaerobic	1			
Mixed	1.02	0.76	1.37	0.886
Anaerobic	0.95	0.67	1.35	0.772

CI, confidence interval.

## Discussion

Results obtained for the 140 athletes who participated in this study confirm the idea that a person's genetic profile is a determining factor for the excretion of testosterone in its glucuronide form (Gallagher et al. 2007b; Strahm et al. 2007). Compared to athletes who were heterozygous for the UGT2B17 gene deletion (ins/del), those with the mutant homozygous genotype (*del*/*del*) showed a mean T/E ratio that was 83% lower, whereas those with the homozygous wt genoptype (*ins*/*ins*) showed a 53% higher value of this ratio. This association is similar to that detected in a study conducted with 116 healthy young Caucasian men, in whom the UGT2B17 gene deletion significantly affected the urinary excretion of androgens without affecting blood hormone levels and this response was most marked in 10 of the 116 subject who were homozygous for the deletion (*del*/*del*) (Juul et al. 2009). Similar steroid excretion modifications have been observed by other authors (Van Renterghem et al. 2010).

In our study, the deletion frequency (*del*/*del*) was 6.9% in Caucasian and 80% in Asian athletes. This frequency is similar to rates reported by other authors (Schulze et al. 2008, 2009), this deletion being extraordinarily common in East Asian countries and relatively rare among Caucasian subjects, affecting 9% of Swedish individuals compared to 67% of Koreans. In our multivariate analysis, the presence of the deletion was the only variable that remained as a significant independent factor with an effect on T/E.

In a study in which athletes were given a 360 mg dose of testosterone and urine T/E levels were monitored over a period of 15 days (Schulze et al. 2009), *del*/*del* individuals showed a lower increase in T/E than athletes with the heterozygous genotype (0.14–5.3 in the *del*/*del* group vs. 1.4–50.4 in *ins*/*del*). In the *ins*/*ins* group, this increase was 2.3–100. Moreover, 40% of the *del*/*del* group athletes failed to return a T/E >4.0 on any of the 15 days.

The results obtained confirm the genetic factor as a determinant element affecting the excretion of testosterone as glucuronide (Jakobsson et al. 2006; Gallagher et al. 2007b; Strahm et al. 2007). According to the World Anti‐Doping Agency (WADA) when a T/E ratio is greater than 4, and there is not additional data from the athlete, a follow‐up process must be activated in order to determine if the atypical result is a consequence of the consumption of testosterone or any of its prohormones. According to our findings a T/E ratio higher than 4, could be perfectly consistent with a natural origin, especially among the wt homozygous (ins/ins) population, therefore the implementation of a genetic study to characterize the UGT2B17 gene deletion would be really useful to avoid the follow‐up and therefore save money and reduce the number of analyses by Gas Chromatography–Combustion–Isotope Ratio Mass Spectrometry (GC‐C‐IRMS) test to determine the ^13^C percentage. Besides the economic consequences (Schulze et al. 2008), the genetic study would allow more accurate interpreting of the longitudinal variations of the T/E ratio and other metabolites of testosterone. In fact, studies have shown that athletes with modifications in the UGT2B17 gene can ingest testosterone and its derivatives without showing a urine T/E ratio as high as 4, or at least very weak modifications (Juul et al. 2009).

It would be interesting for future studies to examine whether the UGT2B17 gene deletion only has an impact on T/E or if other testosterone metabolites are also affected (Ekström et al. 2013), and the response when there is a T administration. Similarly, other metabolization routes (e.g., sulfation) may be promoted in individuals with the deletion mutation. In effect, increased sulfate derivatives of testosterone in subjects with this mutation, though not detected in studies by Borts and Bowers (Juul et al. 2009), were found in other studies to affect 4% of an overall study population of 45 men aged 17–50 years (Schulze et al. 2008) and have been proposed as future markers (Schulze et al. 2011).

Other authors have reported a marked reduction in *β*‐diol metabolites relative to *α*‐diol metabolites in *del*/*del* subjects (Juul et al. 2009; Fabregat et al. 2010).

The main limitation of our study was that being based on an opportunity sample of volunteers, the frequencies of the genotype overall and by ethnical group did not parallel those observed in the general population.

In conclusion, our data indicate that the use of the (T/E) ratio as a marker of T administration needs to be complemented with the UGT2B17 gene deletion characterization in order to have a more accurate perspective of the longitudinal variations of the steroid profile. As recommended by other authors (Sottas et al. 2007; Schulze et al. 2009) a promising approach for detecting abnormal T/E values is the intraindividual study of the steroid profile based on a priori distributions of population factors (Bayesian models). Given the different T/E levels in subjects with the *del*/*del* genotype compared to the other two manifestations of this polymorphism, we propose the correction of T/E values according to the expression form of this mutation.

## Conflict of Interest

None declared.
